# Gestational diabetes detection thresholds and infant growth, nutrition, and neurodevelopment at 12-18 months: a prospective cohort study within a randomized trial

**DOI:** 10.1038/s41372-025-02406-x

**Published:** 2025-09-05

**Authors:** Francesca Amitrano, Komal Manerkar, Jane M. Alsweiler, Cathryn A. Conlon, Caroline A. Crowther, Richard Edlin, Jane E. Harding, Lesley ME McCowan, Michael P. Meyer, Janet A. Rowan, Elaine C. Rush, Christopher JD McKinlay

**Affiliations:** 1 Liggins Institute, University of Auckland, Auckland, New Zealand; 2 Te Whatu Ora, Te Toka Tumai Auckland, Auckland, New Zealand; 3 Department of Paediatrics: Child and Youth Health, University of Auckland, Auckland, New Zealand; 4 School of Sport, Exercise and Nutrition, Massey University, Auckland, New Zealand; 5 Health Systems, University of Auckland, Auckland, New Zealand; 6 Department of Obstetrics and Gynaecology, University of Auckland, Auckland, New Zealand; 7 Te Whatu Ora, Counties Manukau, Auckland, New Zealand; 8 School of Sport and Recreation, Faculty of Health and Environmental Sciences, Auckland University of Technology, Auckland, New Zealand

**Keywords:** Outcomes research, Gestational diabetes

## Abstract

**Objective:**

To assess the impact of gestational diabetes(GDM) detection thresholds on infant growth, nutrition, and neurodevelopment at 12-18 months.

**Design:**

Prospective cohort study within the GEMS trial(ACTRN12615000290594), which randomized pregnant women to detection of GDM using lower or higher glycemic criteria. The main outcomes were overweight/rapid weight gain; food approach appetitive score; energy intake; cognitive z-score.

**Result:**

Compared to control infants, those exposed to GDM detected and treated by higher criteria or by lower but not higher criteria that was untreated, were less likely to have increased overweight/rapid weight gain, possibly with lower energy intake. There were no important differences in appetite and cognition. Infants exposed to GDM by lower but not higher criteria that was treated were similar to controls.

**Conclusion:**

Exposure to treated GDM or untreated GDM detected by lower but not higher criteria, was not associated with increased infant risk factors for obesity or adverse cognitive outcomes.

## Introduction

Gestational diabetes mellitus (GDM) or hyperglycemia first diagnosed in pregnancy is a major public health concern, affecting an estimated 14% of pregnant women globally [1]. GDM is associated with an increased risk of type 2 diabetes for women and obesity and metabolic syndrome in offspring [1]. It may also contribute to neurocognitive impairment and emotional and behavioral difficulties in childhood [2]. Randomized trials have shown that treating GDM with dietary and lifestyle approaches and pharmacological therapy, as appropriate, reduces the risk of large-for-gestational-age infants (LGA >90th centile) by 40–50% [3, 4], but it is unclear whether detection and treatment of GDM reduces the risk of obesity, metabolic disease and adverse neurodevelopment in the next generation.

The Gestational Diabetes Mellitus Trial of Detection Thresholds (GEMS) [5] investigated whether the detection and treatment of GDM in a general obstetric population using the lower diagnostic thresholds set by the International Association of Diabetes in Pregnancy Study Groups (IADPSG) [6], compared to higher criteria in use for over 20 years [7], would decrease the risk of LGA. The use of the IADPSG criteria did not alter the rate of LGA (8.8% vs. 8.9%) but increased the number of inductions (34% vs. 30%) and infants treated for hypoglycemia (11% vs. 8%) [5]. Groups had similar rates of the composite serious outcome (stillbirth, neonatal death, birth trauma, or shoulder dystocia: 2.5% vs. 2.2%), pre-eclampsia (3.7% vs. 3.7%) and neonatal unit admission (4.6% vs. 3.6%), despite more women being diagnosed with GDM (15% vs. 6%). However, in secondary analysis of GEMS participants who met the lower but not higher criteria, the risk of LGA was lower among those who received treatment for GDM compared to those who did not (6.2% vs. 18%) [5].

Late gestation and infancy are a critical period for the establishment of long-term homeostatic set-points and trajectories. For example, increased infant weight gain [8], adipose accrual [8], energy and protein intake [9, 10], and reduced lean mass [11] and duration of breastfeeding [12] have been associated with increased risk of obesity and cardiometabolic disorders later in life. Similarly, late gestation and early infancy are a critical period for the development of brain networks and microstructure [13, 14]. Thus, if the reported associations between GDM and offspring obesity, metabolic disease and adverse neurodevelopment are causal, an association between different severities of GDM and its treatment and infant risk factors for obesity and early developmental progress would be expected. However, there is a paucity of data on the effect of GDM on infant health, especially from clinical trials [15, 16]. We report the results of the BabyGEMS Study at 9 and 12–18 months’ corrected age, a nested cohort study within GEMS, designed to prospectively evaluate the effect of different severities of GDM, both treated and untreated, on infant risk factors for later obesity. We hypothesized that compared to randomly selected control infants without exposure to GDM by lower criteria, infants exposed to GDM detected by higher criteria and infants exposed to GDM by lower (IAPDSG) but not higher criteria that was untreated, but not those exposed to GDM detected by lower (IAPDGS) but not higher criteria that was treated, would have increased overweight or rapid weight gain from birth, higher food approach appetitive trait scores, higher energy intake, and lower cognitive developmental scores.

## Methods

### Participants

The methods of the BabyGEMS Study have been described previously [17]. Briefly, this was a prospective cohort study of infants born to women enrolled in GEMS [5] who met the lower IADPSG diagnostic criteria for GDM [6], regardless of their trial allocation group, and a 5% random sample of control infants whose mothers did not have GDM by the lower criteria (Table 1). In GEMS, women with a singleton pregnancy booked to birth in one of two New Zealand health districts (Auckland City Hospital, Counties Manukau) and no previous diabetes mellitus or GDM, after a one-step 75 g OGTT at 24 to 32 weeks’ gestation, with consent were randomly assigned to diagnosis of GDM using lower or higher criteria. In the Lower Criteria Group, GDM was diagnosed if one or more of the following criteria were met, as recommended by the IADPSG: fasting plasma glucose ≥5.1 mmol/L[ ≥ 92 mg/dL], one-hour plasma glucose ≥10.0 mmol/L[ ≥ 180 mg/dL] or two-hour plasma glucose ≥8.5 mmol/L[ ≥ 153 mg/dL] [18]. In the Higher Criteria Group, GDM was diagnosed with either a fasting plasma glucose ≥5.5 mmol/L[ ≥ 99 mg/dL] or two-hour plasma glucose ≥9.0 mmol/L[162 mg/dL], as recommended by the New Zealand Ministry of Health [7]. Women with OGTT results diagnostic for GDM according to the detection criterion to which they were assigned, were informed that they had the condition and received usual care for GDM, including lifestyle modification, blood glucose monitoring and pharmacologic treatment, as needed. Pharmacologic treatment included metformin and/or insulin, according to national guidance and the preference of the woman and her physician [7]. Women with OGTT results below the assigned detection criterion for GDM, received routine pregnancy care. Numerical OGTT results were not disclosed, such that trial participants, caregivers, and researchers were blinded about the GEMS criteria group allocation, including at follow-up. Women provided written consent at trial entry for assessment of their infants and assent for infant follow-up was confirmed after birth. Maternal ethnicity was determined by self-report, according to national protocols [19]. This study was approved by the Northern B Health and Disability Ethics Committee (13/NTB/18) and was performed in accordance with the Declaration of Helsinki.

**Table 1:** BabyGEMS Study groups.

BabyGEMS group	Controls	Group A	Group B	Group C
Maternal glycemic status	Not GDM by lower criteria: received routine pregnancy care	GDM by lower but not higher criteria: treated. Women received usual treatment for diabetes in pregnancy (GEMS Trial OGTT designated as “GDM”)	GDM by lower but not higher criteria: untreated. Women did not receive treatment for diabetes in pregnancy (GEMS Trial OGTT designated as “not GDM”)	GDM by higher criteria: treated. Women received usual treatment for diabetes in pregnancy (GEMS Trial OGTT designated as “GDM”)
GEMS Trial group	Lower or Higher Criteria Group	Lower Criteria Group	Higher Criteria Group	Lower or Higher Criteria Group
OGTT plasma glucose concentration thresholds mmol/L [mg/dL]	Fasting <5.1 [91.8] and one-hour <10 [180.0] and two-hour <8.5 [153.0]	Fasting ≥5.1 [91.8] to <5.5 [99.0] or two-hour ≥8.5 [153.0] to <9 [162.0] or one-hour ≥10 [180.0]		Fasting ≥5.5 [99.0] or two-hour ≥9 [162.0] (regardless of one-hour value)

*GDM* gestational diabetes mellitus, *IADPSG* International Association of Diabetes and Pregnancy Study Groups, *OGTT* oral glucose tolerance test.

### Assessments

Infants in the BabyGEMS Study were assessed at birth and 5-6 months, as reported previously [17]. At 9 months’ corrected age, parents completed a complementary food frequency questionnaire (CFFQ) to estimate nutritional intake [20, 21], and reported the most recent weight, height, and head circumference recorded in the national child health booklet. At 12–18 months’ corrected age, infants underwent a clinical assessment of growth, nutrition, and development by trained study personnel, including a paediatrician and research dietitians, and midwives. Weight was measured to the nearest 10 g by digital infant scale (SECA 354, Hamburg, Germany); length to the nearest 0.5 cm using an infantometer; head, chest and abdominal circumferences to the nearest 0.1 cm using a non-stretchable lasso tape. The acromio-radial length was measured as the linear distance between left acromiale and radiale to the nearest 1 mm [22]. Subscapular and triceps skinfold thickness was measured twice using Harpenden calipers; if the difference between the first two was >0.4 mm, a third measure was taken, with the median used in analysis. Left arm muscle area (AMA) was calculated from the mid-arm circumference and triceps skinfold thickness [23]. Cognitive development was assessed using the Cognitive Adaptive Test (CAT), a 100-item administered developmental tool, assessing the visual-motor developmental stream and the emergence of problem-solving skills [24]. The CAT has shown to have high positive and moderate negative predictive value for cognitive impairment compared with the Bayley Scales of Infant Development [25]. Parents completed questionnaires about feeding history and nutritional intake (CFFQ), appetitive traits (Children’s Eating Behaviour Questionnaire, CEBQ) [26, 27], and developmental progress in communication, gross motor, and personal-social interaction (Ages and Stages Questionnaires version 3, ASQ-3) [28]. The ASQ-3 is widely used for developmental screening and has a modest agreement with the Bayley Scales of Infant Development [29]. Assessments were conducted from August 2016 to April 2021.

### Outcomes

The pre-specified main outcomes at 12-18 months were overweight or rapid weight gain (BMI z-score >2 or conditional gain in weight z-score from birth >1); food approach appetitive trait score; energy intake; and cognitive z-score. All other assessment measures are reported for completeness.

### Statistical analysis

Statistical analysis was performed with SAS version 9.4 (SAS Institute, Cary, North Carolina, USA). Based on the maternal OGTT results and the treatment that the women received, infants in the BabyGEMS Study were categorized into four glycemic exposure groups: control infants, whose mothers’ OGTT results did not meet the lower criteria and received routine pregnancy care; group A, whose mothers had GDM by lower but not higher criteria and received usual diabetes treatment as they were allocated to the GEMS Lower Criteria Group; group B, whose mothers had GDM by lower but not higher criteria and did not receive treatment for diabetes as they were allocated to the GEMS Higher Criteria Group and were informed that they did not have GDM; and group C, whose mothers had GDM detected using higher criteria and received diabetes treatment, regardless of randomization group (Table 1).

For analysis, ethnicity was prioritized in the following order: Māori, Pacific, Indian, Other Asian, Other non-European, and European [19]. Anthropometric data were converted to gestation- and sex-specific z-scores, using the World Health Organization growth standards for weight, height, BMI, head circumference, skinfold thickness, and arm circumference [30] and the CDC standards for AMA [31]. Cognitive scores were converted to age-specific z-scores from normative data [32], and infants who could not complete the CAT due to an underlying neurological condition were assigned a z-score of -3. Conditional growth was computed as the residuals from sex-specific regression of size (z-score) on size at the previous assessment point [33].

In the primary analysis, the outcomes of infants in the three GDM exposure groups were compared with control infants using generalized linear models appropriate for the dependent variable, with Dunnett correction of family-wise error rate. Models were adjusted for potential confounding by maternal body size (BMI at <18 weeks’ gestation), New Zealand Deprivation Index 2013 [34], maternal ethnicity, and infant sex. Adjusted exposure effects are presented as risk difference (aRD) or mean difference (aMD) with 95% confidence intervals (CI). For categorical variables, adjusted relative effect (odds ratio, aOR) was also estimated with 95% CI. Missing outcome data were not imputed.

## Results

Of 4061 participants enrolled in GEMS, 804 infants were eligible for inclusion in the BabyGEMS Study, of whom 760 participated [17]. After birth, 30 participants withdrew, leaving 730 infants eligible for inclusion in the follow-up at 12-18 months. A total of 620 infants (85% of eligible) were assessed, including 135 control infants and 485 infants exposed in utero to GDM (Supplementary Fig. S1). Compared to the infants assessed at 12 months, those not assessed were born to mothers more likely to be of Māori and Pacific ethnicity, with higher BMI, lower socioeconomic status, and a history of chronic hypertension (Supplementary Table S1).

At trial entry, mothers of BabyGEMS infants who had GDM detected and treated using higher criteria (group C), compared with the control group, had higher BMI, shorter stature, and were more likely to be nulliparous and have a family history of diabetes mellitus (Table 2). While the ethnicity of women with GDM was not significantly different to control women, among those with GDM detected and treated using higher criteria (group C), there was a preponderance of women of Indian ethnicity (Table 2). Moreover, women in this group treated for GDM, compared with control women, had less gestational weight gain (Table 2).

**Table 2:** Baseline characteristics of infants, and their mothers, in the BabyGEMS Study at 12 to 18 months’ corrected age.

	Control	Group A GDM by lower but not higher criteria: treated	Group B GDM by lower but not higher criteria: untreated	Group C GDM by higher criteria: treated
	*N* = 135	*N* = 154	*N* = 146	*N* = 185
Maternal pregnancy characteristics				
Age at GEMS Trial entry–year	31.5 (5.0)	32.5 (5.2)	32.6 (5.5)	31.9 (4.7)
Gestational age at GEMS Trial entry–weeks	21.6 (4.8)	22.1 (4.3)	22.7 (4.7)	22.5 (4.6)
BMI–kg/m^2^	26.6 (6.7)	28.4 (6.4)	28.3 (6.4)	28.7 (7.7)*
<25.0	69 (51%)	52 (34%)	51 (35%)	61 (33%)
25.0 to 29.9	30 (22%)	49 (32%)	46(32%)	59 (32%)
≥30.0	36 (27%)	53 (34%)	49 (34%)	64 (35%)
Maternal height–cm	163.9 (7.6)	162.1 (6.6)	163.1 (7.8)	160.8 (6.3)*
Nulliparous	55 (41%)	71 (46%)	62 (42%)	103 (56%)*
Prioritized ethnicity^a^				
Māori	11 (8%)	12 (8%)	14 (10%)	12 (7%)
Pacific	21 (16%)	17 (11%)	26 (18%)	32 (17%)
Indian	14 (10%)	35 (23%)	25 (17%)	57 (31%)
Other Asian	23 (17%)	33 (21%)	30 (21%)	43 (23%)
Other non-European	8 (6%)	14 (9%)	8 (5%)	11 (6%)
European	58 (43%)	43 (28%)	43 (29%)	29 (16%)
Lower socioeconomic status	46 (34%)	64 (42%)	65 (45%)	78 (42%)
Smoker at GEMS Trial entry	3 (2%)	5 (3%)	5 (3%)	8 (4%)
Recruitment site				
Counties Manukau	36 (27%)	54 (35%)	59(40%)	73 (40%)
Auckland City	89 (66%)	98 (64%)	80 (55%)	109 (59%)
History of chronic hypertension	4 (3%)	6 (4%)	5 (3%)	9 (5%)
Pregnancy induced hypertension	6 (4%)	1 (1%)	8 (5%)	10 (5%)
Family history of diabetes	47 (35%)	72 (47%)	55 (38%)	109 (59%)*
Gestational weight gain—kg	12.4 (8.1)	10.0 (6.5)	11.9 (10.2)	9.6 (7.6)*
OGTT plasma glucose concentration at GEMS Trial entry^c^				
Fasting—mmol/L	4.3 (0.3)	4.8 (0.4)*	4.8 (0.4)*	5.2 (0.9)*
1 h—mmol/L	6.9 (1.4)	9.5 (1.4)*	9.4 (1.7)*	10.4 (1.8)*
2 h—mmol/L	5.7 (1.3)	7.4 (1.2)*	7.4 (1.1)*	9.5 (1.8)*
Pharmacologic treatment ^b^				
Metformin	0 (0%)	55 (36%)*	2 (1%)	58 (32%)*
Insulin	0 (0%)	19 (12%)*	1 (1%)	37 (20%)*
Metformin and insulin	0 (0%)	27 (18%)*	0 (0%)	40 (22%)*
None	135 (100%)	53 (34%)*	143 (98%)	49 (27%)*
Induction of labour	34 (25%)	93 (60%)*	46 (32%)	102 (55%)*
Caesarean delivery	49 (36%)	63 (41%)	70 (48%)	70 (38%)
Emergency caesarean	26 (19%)	42 (27%)	45 (31%)	49 (27%)
Infant characteristics				
Sex–female	58 (43%)	76 (49%)	73 (50%)	86 (47%)
Gestational age at birth–weeks	39.6 (1.3)	38.7 (1.1)*	39.2 (1.5)*	38.2 (1.3)*
37 to 38 weeks	35 (26%)	75 (49%)*	45 (31%)	112 (61%)*
<37 weeks	3 (2%)	9 (6%)	6 (4%)	20 (11%)*
Apgar score <7 at 5 minutes	5 (4%)	1 (1%)	3 (2%)	1 (1%)
Age at follow-up—months	12.5 (1.4)	12.3 (1.1)	12.5 (1.0)	12.4 (1.5)

Data are mean (standard deviation) or number (percent). * *P* < 0.05 for comparison with Controls. Lower socioeconomic status defined as New Zealand Deprivation Index 8–10. Maternal BMI calculated from pre-pregnancy weight or if this was unavailable, from weight at pregnancy booking.

*BMI* body mass index, *GEMS* Gestational Diabetes Mellitus Trial of Detection Thresholds.

^a^Maternal ethnicity was determined by self-report, according to national protocols, and prioritised for analysis as Māori, Pacific, Indian, Other Asia, Other non-European, European.

^b^Three women in Group B (2%) received a late diagnosis of GDM at ≥35 weeks’ gestation following a clinically indicated non-GEMS OGTT and commenced pharmacologic treatment.

^c^To convert glucose concentration in mmol/L to mg/dL divide by 0.0555.

Compared with the control group, infants exposed to GDM were similar for sex, cesarean birth, and low Apgar score. However, infants exposed to GDM, treated or untreated, had a shorter gestation than controls. Infants exposed to GDM that was treated (groups A and C) were more likely than controls to be born after induction of labor and at early term (37–38 weeks), and infants exposed to GDM detected and treated by higher criteria (group C) were also more likely to be born preterm (<37 weeks) (Table 2).

### Main outcomes

Compared with the control group, there was some evidence that infants exposed to GDM detected by lower but not higher criteria that was untreated (group B) and infants exposed to GDM detected by higher criteria and treated (group C) may have been less likely to present with overweight or rapid weight gain at 12-18 months (controls 28%; group B 13%, aRD −15% 95%CI −30,3, aOR 0.3, 95% CI 0.1,0.7; group C 15%, aRD −14% 95%CI −27, 2, aOR 0.4, 95% CI 0.2,0.8; Table 3). This appeared to be related to reductions in both high BMI and rapid weight gain from birth, and may have been related to reduced energy intake (Table 3). In infants exposed to GDM detected by lower but not higher criteria that were treated (group A), the rate of overweight or rapid weight gain at 12 months was similar to control infants (controls 28%; group A 22%, aRD −6% 95%CI −20,6, aOR 0.6, 95%CI 0.3,1.3; Table 3). Compared with control infants, those exposed to GDM, treated or untreated, did not show any important differences at 12–18 months in food approach appetitive trait score or cognitive z-score, regardless of GDM detection criteria and treatment (Table 3).

**Table 3:** Main outcomes at 12 to 18 months’ corrected age.

	Control	*N*	Group A GDM by lower but not higher criteria: treated	*N*	Adjusted RD or MD (95% CI) *OR [95% CI]*	Group B GDM by lower but not higher criteria: untreated	*N*	Adjusted RD or MD (95% CI) *OR [95% CI]*	Group C GDM by higher criteria treated	*N*	Adjusted RD or MD (95% CI) *OR [95% CI]*
Composite of infant overweight or rapid weight gain from birth^a^	35 (28%)	123	32 (22%)	147	−6 (−20, 6) *0.6 [0.3, 1.3]*	18 (13%)	134	−15 (−30, 3) *0.3 [0.1, 0.7]*	26 (15%)	174	−14 (−27, 2) *0.4 [0.2, 0.8]*
Infant overweight	24 (20%)	123	14 (10%)	147	−10 (−25, 5) *0.3 [0.1, 0.9]*	8 (6%)	134	−14 (−28, 2) *0.2 [0.1, 0.5]*	9 (5%)	174	−15 (−29, 1) *0.1 [0.0, 0.4]*
Rapid gain in weight z-score from birth	27 (22%)	123	28 (19%)	147	−3 (−16, 9) *0.8 [0.4, 1.6]*	14 (10%)	134	−12 (−25, 2) *0.4 [0.2, 0.9]*	22 (13%)	174	−9 (−21, 3) *0.4 [0.2, 0.9]*
CEBQ											
Food approach subscale	8.3 (1.7)	123	8.2 (1.8)	144	−0.1 (−0.6, 0.4)	8.4 (1.6)	140	−0.1 (−0.6, 0.4)	8.5 (1.7)	174	0.0 (−0.5, 0.5)
CFFQ											
Energy—kJ/kg/day	3490 (2246)	107	2957 (1410)	135	−490 (−1030, 50)	3120 (1625)	125	−383 (−929, 163)	2991 (1914)	154	−499 (−1042, 43)
Protein—percent energy	17 (6)	107	17 (5)	135	0.4 (−1.2, 2.0)	17 (5)	125	0.5 (−1.1, 2.2)	16 (5)	154	0.6 (−1.1, 2.2)
Fat—percent energy	31 (8)	107	32 (7)	135	0.4 (−1.8, 2.7)	31 (7)	125	0.2 (−2.1, 2.4)	31 (8)	154	0.1 (−2.1, 2.4)
Carbohydrate—percent energy	50 (9)	107	49 (8)	135	−1.0 (−3.5, 1.5)	50 (8)	125	−0.7 (−3.2, 1.9)	51 (9)	154	0.9 (−3.4, 1.7)
CAT											
Cognitive z-score	0.27 (1.20)	121	0.01 (0.98)	143	−0.16 (−0.46, 0.15)	0.22 (1.06)	131	−0.03 (−0.28, 0.33)	0.30 (0.97)	171	0.16 (-0.14, 0.46)

Data are mean (standard deviation) or number (percent). See Table 1 for oral glucose tolerance test detection criteria associated with each group. Analyses adjusted for potential confounding by maternal body size (BMI), socioeconomic status (New Zealand Deprivation Index), ethnicity and infant sex, with Dunnett correction of family-wise error rate.

*CEBQ* Child Eating Behaviour Questionnaire, *CFFQ* Complementary Food Frequency Questionnaire, *CAT* Cognitive Adaptive Test, *CI* confidence interval, *OR* odds ratio, *MD* mean difference, *RD* risk difference.

^a^Overweight defined as BMI z-score >2 and rapid weight gain from birth to 12-18 months as conditional gain in weight z-score for weight and length z-score at birth >1.

### Additional outcomes

Consistent with the main finding for overweight or rapid weight gain, infants exposed to GDM detected by higher criteria and treated (group C), compared with control infants, had lower z-scores for weight and BMI at 12−18 months (weight z-score aMD −0.39, 95%CI −0.69, −0.09; Table 4). This did not appear to be associated with any important differences in skin fat or muscle mass, as z-scores for skinfold thickness and arm muscle area were similar to the control group (Table 4). There was some evidence that infants in group C may have had altered skeletal size and/or organ mass, compared with control infants, with slightly shorter post-axial length (acromioradiale aMD −0.4 cm, 95%CI −0.8,0.0), smaller head circumference (z-score aMD −0.37, 95% CI −0.70, −0.04), reduced chest circumference (aMD −0.7 cm, 95%CI −1.4,0.0), and possibly shorter axial length (z-score AMD -0.25, 95%CI −0.57,0.06) (Table 4). Also consistent with the main finding, growth in weight from birth to 12−18 months, compared with control infants, was reduced (conditional z-score gain aMD −0.40, 95% CI −0.66, −0.12), although slowing of growth after 6 months was less apparent (conditional z-score gain from 6 months aMD −0.20, 95%CI −0.47,0.11; weight z-score at 9 months aMD −0.58, 95%CI −1.18,0.03; Supplementary Table S3). A similar pattern of results for these additional anthropometric and growth outcomes was seen for infants exposed to GDM detected by lower but not higher thresholds that were untreated (group B) (Table 4, Supplementary Table S3).

**Table 4:** Body size, composition, and growth at 12 to 18 months’ corrected age.

	Control	*N*	Group A GDM by lower but not higher criteria: treated	*N*	Adjusted MD (95% CI)	Group B GDM by lower but not higher criteria: untreated	*N*	Adjusted MD (95% CI)	Group C GDM by higher criteria treated	N	Adjusted MD (95% CI)
Body size											
Length—cm	76.5 (3.2)	123	76.0 (3.4)	147	−0.3 (−1.2, 0.5)	75.8 (3.2)	135	−0.6 (−1.5, 0.2)	75.3 (3.4)	175	−1.0 (−1.8, −0.1)
Weight—kg	10.31 (1.41)	123	9.96 (1.43)	147	−0.24 (−0.60, 0.13)	9.92 (1.31)	136	−0.38 (−0.74, −0.01)	9.67 (1.41)	175	−0.54 (−0.90, −0.18)
BMI—kg/m^2^	17.6 (1.8)	123	17.3 (1.8)	146	−0.3 (−0.7, 0.2)	17.2 (1.5)	134	−0.4 (−0.9, 0.1)	17.0 (1.7)	174	−0.5 (−1.0, 0.0)
Head circumference—cm	46.5 (1.7)	123	46.1 (1.6)	146	−0.1 (−0.6, 0.3)	46.1(1.7)	133	−0.2 (−0.7, 0.2)	45.7 (1.7)	175	−0.6 (−1.0, −0.1)
Abdominal circumference—cm	45.6 (3.5)	123	45.4 (3.6)	145	0.0 (−1.0, 0.9)	44.8 (3.4)	133	−0.8 (−1.7, 0.2)	44.4 (3.4)	174	−0.9 (−1.8, 0.1)
Chest circumference—cm	47.0 (2.4)	122	46.7 (2.8)	145	−0.1 (−0.8, 0.6)	46.3 (2.7)	133	−0.7 (−1.4, 0.0)	46.1 (2.4)	174	−0.7 (−1.4, 0.0)
Arm circumference—cm	15.4 (1.6)	123	15.3(1.4)	146	−0.1 (−0.5, 0.4)	15.0(1.6)	132	−0.4 (−0.9, 0.0)	15.0 (1.5)	174	−0.3 (−0.8, 0.1)
Acromio-radiale—cm	13.4 (1.3)	123	12.9 (1.4)	146	−0.4 (−0.8, 0.0)	13.1 (1.4)	132	−0.3 (−0.6, 0.1)	13.0 (1.2)	174	−0.4 (−0.8, 0.0)
Z-scores for age and sex											
Length	0.37 (1.15)	123	0.27 (1.16)	147	−0.01 (−0.33, 0.32)	0.15 (1.12)	135	−0.20 (−0.52, 0.13)	0.01 (1.23)	175	−0.25 (−0.57, 0.06)
Weight	0.65 (1.14)	123	0.47 (1.14)	147	−0.12 (−0.43, 0.18)	0.39 (1.06)	136	−0.28 (−0.59, 0.02)	0.18 (1.19)	175	−0.39 (−0.69, −0.09)
BMI	0.60 (1.17)	123	0.43 (1.16)	146	−0.17 (−0.48, 0.15)	0.42 (1.00)	134	−0.23 (−0.54, 0.09)	0.24 (1.19)	174	−0.33 (−0.64, −0.02)
Head circumference	0.57 (1.25)	123	0.45 (1.17)	146	−0.07 (−0.40, 0.27)	0.42 (1.20)	133	−0.13 (−0.47, 0.20)	0.08 (1.19)	175	−0.37 (−0.70, −0.04)
Arm circumference	0.75 (1.33)	123	0.67 (1.18)	146	−0.03 (−0.38, 0.33)	0.40 (1.35)	132	−0.36 (−0.72, 0.00)	0.39 (1.27)	174	−0.26 (−0.61, 0.09)
Body composition											
Subscapular skinfold—mm	7.7 (1.8)	122	7.9 (2.2)	144	0.2 (−0.4, 0.8)	7.8 (1.9)	134	0.1 (−0.4, 0.7)	7.6 (1.9)	171	0.0 (−0.5, 0.6)
Triceps skinfold—mm	10.2 (3.0)	122	10.1 (2.8)	144	0.1 (−0.6, 0.9)	9.6 (2.3)	134	−0.4 (−1.2, 0.3)	9.7 (2.4)	171	−0.1 (−0.9, 0.6)
Arm muscle cross-sectional area—cm^2^	12.1 (2.9)	122	11.8 (2.6)	144	−0.2 (−1.0, 0.5)	11.6 (2.9)	132	−0.6 (−1.4, 0.2)	11.5 (2.6)	171	−0.6 (−1.3, 0.2)
Z-scores for age and sex											
Subscapular skinfold	0.75 (1.12)	122	0.77 (1.31)	144	0.05 (−0.30, 0.40)	0.81 (1.14)	134	0.06 (−0.29, 0.41)	0.64 (1.23)	171	−0.04 (−0.38, 0.31)
Triceps skinfold	0.96 (1.40)	122	0.92 (1.34)	144	0.08 (−0.29, 0.45)	0.74 (1.23)	134	−0.15 (−0.53, 0.22)	0.74 (1.27)	171	−0.03 (−0.39, 0.34)
Arm muscle area	−0.21 (1.24)	122	−0.19 (0.98)	144	0.01 (−0.31, 0.33)	−0.38 (1.14)	132	−0.20 (−0.52, 0.12)	−0.40 (1.13)	171	−0.16 (−0.48, 0.15)
Conditional gain in z-score											
Length z-score from 6 months	0.04 (0.99)	123	0.07 (1.00)	147	0.04 (−0.23,0.36)	−0.03 (0.95)	135	−0.06 (−0.36, 0.24)	−0.06 (1.05)	175	−0.09 (−0.36,0.23)
Weight z-score from 6 months^a^	0.18 (0.98)	123	0.04 (1.01)	147	−0.10 (−0.35, 0.23)	−0.06 (0.94)	136	−0.19 (−0.47, 0.11)	−0.11 (1.05)	175	−0.20 (−0.47, 0.11)
Weight z-score from birth^b^	0.24 (1.00)	123	0.12 (0.99)	147	−0.10 (−0.37, 0.18)	−0.14 (0.94)	134	−0.39 (−0.68, −0.13)	−0.17 (1.01)	174	−0.40 (−0.66, −0.12)

Data are mean (standard deviation). See Table 1 for oral glucose tolerance test diagnostic criteria associated with each group. BMI, body mass index. Analyses adjusted for potential confounding by maternal body size (BMI), socioeconomic status (New Zealand Deprivation Index), ethnicity and infant sex, with Dunnett correction of family-wise error rate.

^a^Conditional growth in weight z-score for weight z-score and length z-score at 5–6 months.

^b^Conditional growth in weight z-score for weight and length z-score at birth.

There was some evidence that infants exposed to GDM detected by lower but not higher thresholds that were treated (group A) may have been less likely than the control group to have rapid weight gain from birth (aMD −10, 95%CI −25,5; aOR 0.3, 95%CI 0.1,0.9), but there were no important differences in other additional anthropometric and growth outcomes.

For other additional outcomes, infants exposed to GDM, treated or untreated, were similar to control infants, including language, gross motor and personal-social development at 12–18 months (Table 5); feeding at 9 and 12-18 months; energy and macronutrient intake at 9 months (Supplementary Table S4); and other appetitive traits at 12–18 months (Supplementary Table S2).

**Table 5:** Neurodevelopment at 12 to 18 months’ corrected age.

	Control	*N*	Group A GDM by lower but not higher criteria: treated	*N*	Adjusted RD or MD (95% CI) *OR [95% CI]*	Group B GDM by lower but not higher criteria: untreated	*N*	Adjusted RD or MD (95% CI) *OR [95% CI]*	Group C GDM by higher criteria treated	*N*	Adjusted RD or MD (95% CI) *OR [95% CI]*
*(CAT)*											
Low cognitive score	10 (8%)	121	19 (13%)	143	4 (−8, 15) *1.5 [0.5, 4.0]*	14 (11%)	131	2 (−10, 12) *1.1 [0.4, 3.2]*	12 (7%)	171	−2 (−15, 9) *0.7 [0.2, 1.9]*
*(ASQ-3)*											
Communication score	46 (13)	122	49 (11)	147	2.3 (−1.0, 5.5)	49 (11)	138	2.7 (−0.6, 6.0)	50 (11)	175	2.5 (−0.7, 5.8)
Low communication score	24 (20%)	122	16 (11%)	147	−6 (−20, 7) *1.7 [0.7, 4.1]*	14 (10%)	138	−9 (−20, 7) *1.9 [0.8, 4.5]*	13 (7%)	175	−11 (−22, 5) *2.4 [1.0, 6.1]*
Gross motor score	45 (18)	122	44 (18)	147	−2.3 (−6.9, 2.2)	48 (15)	138	1.6 (−2.9, 6.2)	48 (14)	175	0.6 (−3.9, 5.1)
Low gross motor score	27 (22%)	122	39 (27%)	147	7 (−5, 20) *0.7 [0.3, 1.3]*	31 (22%)	138	3 (−6, 18) *0.8 [0.4, 1.7]*	36 (21%)	175	−2 (−16, 8) *0.8 [0.4, 1.7]*
Personal-social score	42 (14)	122	41 (15)	147	−1.1 (−5.2, 3.0)	45 (14)	139	2.7 (−1.3, 6.8)	43 (15)	175	0.4 (−3.6, 4.5)
Low personal-social score	31 (25%)	122	40 (27%)	147	2 (−13, 12) *0.9 [0.5, 1.8]*	24 (17%)	139	−10 (−22, 3) *1.6 [0.8, 3.4]*	45 (26%)	175	1 (−12, 13) *1.0 [0.5, 1.9]*

Data are mean (standard deviation) or number (percent). ASQ low scores defined as 1 or 2 standard deviations below the test mean. Low cognitive scores defined as 1 standard deviation below the test mean. Analyses adjusted for potential confounding by maternal body size (body mass index), socioeconomic status (New Zealand Deprivation Index), ethnicity and infant sex, with Dunnett correction of family-wise error rate.

*ASQ-3* Ages and Stages Questionnaire, version 3, *MD* mean difference, *OR* odds ratio, *RD* risk difference, *CAT* Cognitive Adaptive Test.

## Discussion

Contrary to the study hypothesis, at 12–18 months’ corrected age, compared to control infants, those exposed to GDM detected and treated by higher criteria (group C) and those exposed to GDM by lower (IAPDSG) but not higher criteria that was untreated (group B), were less likely to have increased overweight or rapid weight gain from birth, possibly related to lower energy intake, but there were no important differences in the main appetite and neurocognitive outcomes. Infants exposed to GDM by lower (IADPSG) but not higher criteria that was treated (group A) were similar to control infants for these outcomes, as hypothesized.

Several studies have shown an association between exposure to GDM and overweight/obesity in later childhood and adolescence [35–37], although it remains unclear whether maternal treatment of GDM alters this risk [1, 38]. Wider epidemiological evidence has related childhood obesity to altered infant growth and nutrition, including rapid weight gain [8] and adipose accrual [8]; higher energy and protein intake [9, 10]; more vigorous feeding styles [39]; and a shorter duration of breastfeeding [12]. Thus, we hypothesized that these factors would be likely mediators of any association between GDM and later obesity, and that infants exposed to greater degrees of maternal dysglycemia would be more likely to display these traits than control infants. The reason that our study showed opposite findings is unclear, but it is possible that the observed “catch-down” growth in groups B and C may represent a physiological compensation to altered growth in utero. This finding is not unique; for example, GDM exposure has been previously associated with lower weight and length z-scores at 2 weeks [40] and 12 months of age [41]. There was some evidence in the present study that slower weight gain was associated with lower energy intake, but energy intake did not have any obvious correlation with feeding and appetitive behavior. It remains possible that differences in infant growth relate to intrinsic changes in metabolism and regulation of growth. Overall, the BabyGEMS Study emphasizes the importance of conducting prospective evaluation at multiple time points throughout childhood to fully understand the impact of perinatal exposures on fetal and postnatal growth and body composition.

Although GDM has been associated with neurodevelopmental impairment in offspring [2, 42, 43], it remains unclear to what extent this is influenced by confounder bias, as GDM is also associated with maternal obesity, lower socioeconomic status and preterm or early-term birth, all of which are associated with worse cognitive function in childhood [44, 45]. The BabyGEMS Study provided an opportunity to assess the effect of GDM, treated or untreated, on early development within the context of a randomized trial, including contemporaneous, randomly selected, prospective population control infants not exposed to GDM, thereby minimizing confounding. Within this design, we did not observe any adverse effects of exposure to treated GDM or untreated GDM detected by lower but not higher criteria GDM exposure on neurodevelopmental outcomes at 12–18 months. These findings are consistent with other recent cohort studies that also did not find any association between GDM and cognitive, language, motor, or behavioral function at 18-36 months [46] or school-age [47]. Taken together, these data suggest that the risk factors for GDM are likely to have a larger influence on early brain development than maternal dysglycemia per se. However, follow-up data are awaited from several large GDM trials to determine the risks and benefits of tighter maternal treatment targets [48] or earlier commencement of GDM therapy [49] on childhood neurodevelopmental outcomes.

### Strengths and limitations

The strengths of this study are its prospective design and nesting within a clinical trial, such that treatment for less severe GDM was effectively randomized, minimizing confounding. Moreover, control infants were randomly selected from the general obstetric population and prospectively followed within GEMS, reducing bias from regression to the mean [50]. In addition, the cohort was demographically diverse, such that results are likely to be applicable to other populations.

This study has several limitations. First, for ethical reasons, it was not possible to include an untreated higher criteria group, making the interpretation of the results for group C more challenging. In particular, it is difficult to determine whether the anthropometric differences shown by infants in this group compared with Controls were related to the maternal glycemic environment or the treatment the mothers received. Second, body composition measures could only be assessed through anthropometry, without any direct measures of soft tissue mass, because of the challenges of measuring body composition at this age in large field studies [51]. Finally, since GEMS was a pragmatic trial, data on maternal glycemic control were not available. Therefore, the actual fetal glycemic exposure can only be inferred from the maternal detection and treatment thresholds.

## Conclusion

Exposure to treated GDM or untreated GDM detected by lower but not higher criteria was not associated with increased infant risk factors for later obesity or adverse cognitive outcomes at 12–18 months’ corrected age. Exposure to treated gestational diabetes (GDM) or untreated GDM detected by lower but not higher criteria was not associated with increased infant risk factors for later obesity or adverse cognitive outcomes at 12–18 months’ corrected age.

## Supplementary information

Supplementary material

## Data Availability

The datasets generated and/or analyzed during the current study are not publicly available as ethical approval for this study did not include sharing of individual data.

## Code availability

The SAS analysis code is available by contacting the corresponding author.
