# Supplementary material for: Gestational diabetes detection thresholds and infant growth, nutrition, and neurodevelopment at 12-18 months: a prospective cohort study within a randomized trial
Source: J Perinatol. 2025 Sep 5;45(10):1360–9. doi: 10.1038/s41372-025-02406-x (PMC12479360; doi:10.1038/s41372-025-02406-x)
Supplement: Supplementary file 1 — Supplementary material [file 41372_2025_2406_MOESM1_ESM.docx]

**Supplementary Data**

Gestational diabetes detection thresholds and infant growth, nutrition, and neurodevelopment at 12-18 months: a prospective cohort study within a randomised trial. Francesca Amitrano, Komal Manerkar, Jane M Alsweiler, Cathryn A Conlon, Caroline A Crowther, Richard Edlin, Jane E Harding, Lesley ME McCowan, Michael P Meyer, Janet A Rowan, Elaine C Rush, Christopher JD McKinlay.

Contents

[Figure S1. Participant flow in the BabyGEMS Study at 12 to 18 months. 2](#_Toc184315813)

[Table S1. Baseline characteristics of infants enrolled in the BabyGEMS Study, and their mothers, who were and were not assessed at 12 to 18 months. 3](#_Toc184315814)

[Table S2. Feeding and appetitive traits at 12 to 18 months. 4](#_Toc184315815)

[Table S3. Anthropometric measures at 9 months. 5](#_Toc184315816)

[Table S4. Feeding and nutritional intake at 9 months. 6](#_Toc184315817)

#
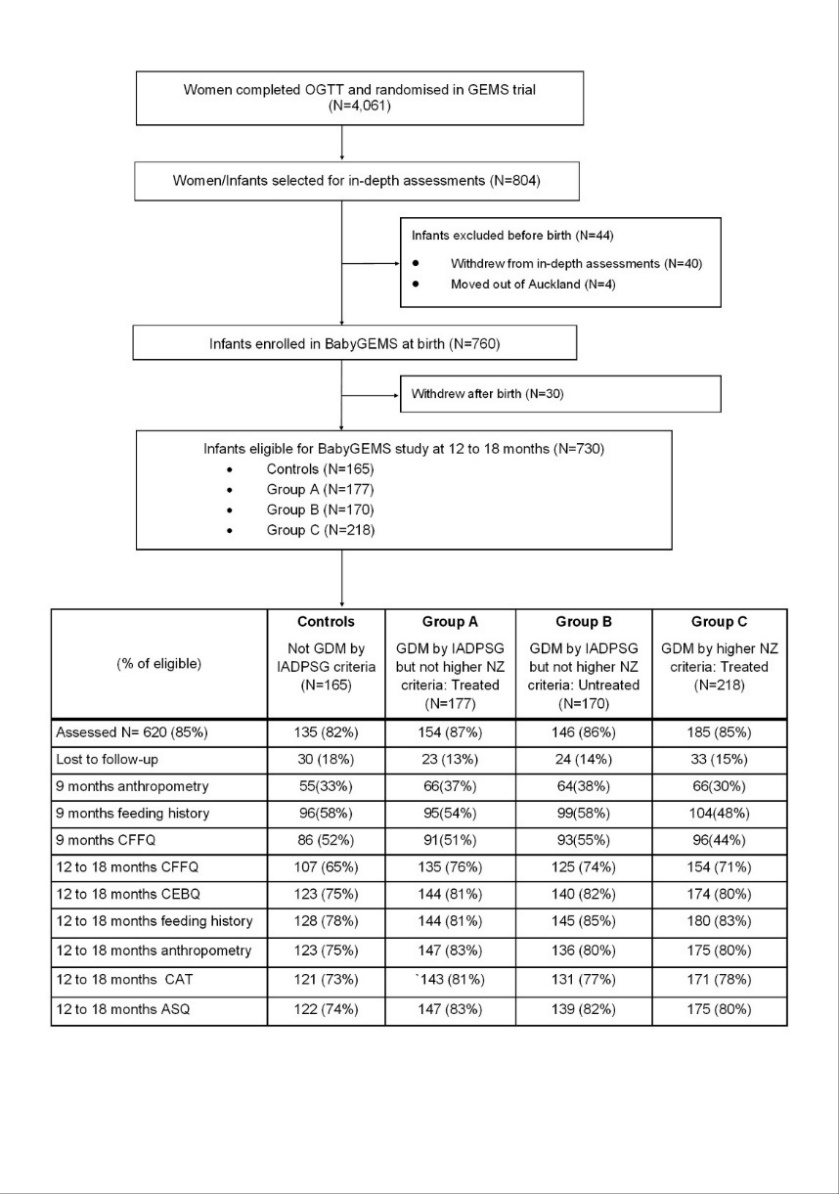
Figure S1. Participant flow in the BabyGEMS Study at 12 to 18 months.

# Table S1. Baseline characteristics of infants enrolled in the BabyGEMS Study, and their mothers, who were and were not assessed at 12 to 18 months.

| Characteristics | Assessed | N | Not Assessed | N | P |
| --- | --- | --- | --- | --- | --- |
| *Maternal pregnancy characteristics* |  |  |  |  |  |
| Age at GEMS Trial entry–year | 32.2 (5.1) | 620 | 31.2 (5.0) | 110 | 0.08 |
| Gestational age at GEMS Trial entry–weeks | 22.2 (4.6) | 620 | 22.5 (3.5) | 110 | 0.64 |
| BMI–kg/m^2^ | 28.1 (6.9) | 620 | 29.6 (7.8) | 110 | 0.04 |
| <25.0 | 233 (38%) |  | 32 (29%) |  |  |
| 25.0 to 29.9 | 184 (30%) |  | 36 (33%) |  |  |
| ≥30.0 | 203 (33%) |  | 42 (38%) |  |  |
| Maternal height–cm | 162.3 (7.1) | 620 | 163.1 (7.6) | 110 | 0.30 |
| Nulliparous | 291 (47%) | 620 | 45 (41%) | 110 | 0.24 |
| Prioritized ethnicity† |  | 620 |  | 110 | <0.001 |
| Māori | 49 (8%) |  | 15 (14%) |  |  |
| Pacific | 97 (16%) |  | 27 (25%) |  |  |
| Indian | 131 (21%) |  | 31 (28%) |  |  |
| Other Asian | 129 (21%) |  | 11(10%) |  |  |
| Other non-European | 41 (7%) |  | 10 (9%) |  |  |
| European | 173 (28%) |  | 16 (15%) |  |  |
| Lower socioeconomic status | 254 (41%) | 620 | 60 (55%) | 110 | 0.01 |
| Smoker at GEMS Trial entry | 21 (3%) | 620 | 7 (6%) | 110 | 0.14 |
| Recruitment site |  | 620 |  | 110 |  |
| Counties Manukau | 223 (36%) |  | 47 (43%) |  |  |
| Auckland City | 376 (61%) |  | 58 (53%) |  |  |
| History of chronic hypertension | 25 (4%) | 620 | 10 (9%) | 110 | 0.03 |
| Pregnancy induced hypertension | 25 (4%) | 620 | 6 (5%) | 110 | 0.50 |
| Family history of diabetes | 284 (46%) | 620 | 45 (41%) | 110 | 0.34 |
| Gestational weight gain—kg | 10.8 (8.2) | 620 | 12.8 (12.2) | 110 | 0.18 |
| OGTT plasma glucose concentration at GEMS Trial entry‡ |  |  |  |  |  |
| Fasting—mmol/L | 4.8 (0.7) | 620 | 4.9 (0.7) | 110 | 0.40 |
| 1 hour—mmol/L | 9.2 (2.0) | 620 | 9.2 (1.9) | 110 | 0.97 |
| 2 hours—mmol/L | 7.7 (2.0) | 620 | 7.5 (1.9) | 110 | 0.55 |
| Pharmacologic treatment |  | 620 |  | 110 | 0.78 |
| Metformin | 115 (19%) |  | 17 (15%) |  |  |
| Insulin | 57 (9%) |  | 10 (9%) |  |  |
| Metformin and insulin | 68 (11%) |  | 15 (14%) |  |  |
| None | 380 (61%) |  | 68 (62%) |  |  |
| Induction of labour | 275 (44%) | 620 | 45 (41%) | 110 | 0.50 |
| Caesarean delivery | 252 (41%) | 620 | 39 (35%) | 110 | 0.31 |
| Emergency caesarean | 162 (26%) | 620 | 25 (23%) | 110 | 0.45 |
| *Infant characteristics* |  |  |  |  |  |
| Sex–female | 294 (47%) | 620 | 48 (44%) | 110 | 0.46 |
| Gestational age at birth–week | 38.9 (1.4) | 620 | 38.5 (2.0) | 110 | 0.02 |
| 37 to 38 weeks | 267 (43%) | 620 | 53 (48%) | 110 | 0.32 |
| <37 weeks | 39 (6%) | 620 | 11 (10%) | 110 | 0.16 |
| Apgar score <7 at 5 minute | 10 (2%) | 620 | 4 (4%) | 110 | 0.17 |

Data are mean (standard deviation) or number (percent). BMI, body mass index; GEMS, Gestational Diabetes Mellitus Trial of Detection Thresholds; NZ, New Zealand. Lower socioeconomic status defined as New Zealand Deprivation Index 8 to 10. Maternal BMI calculated from pre-pregnancy weight or if this was unavailable, from weight at pregnancy booking. ^†^ Maternal ethnicity was determined by self-report, according to national protocols, and prioritised for analysis as Māori, Pacific, Indian, Other Asia, Other non-European, European. ‡To convert glucose concentration in mmol/L to mg/dL divide by 0.0555.

# Table S2. Feeding and appetitive traits at 12 to 18 months.

|  | Control | N | Group A  GDM by lower but not higher criteria: treated | N | Adjusted RD  (95% CI)  *OR [95% CI]* | Group B  GDM by lower but not higher criteria: untreated | N | Adjusted RD  (95% CI)  *OR [95% CI]* | Group C  GDM by higher criteria treated | N | Adjusted RD  (95% CI)  *OR [95% CI]* |
| --- | --- | --- | --- | --- | --- | --- | --- | --- | --- | --- | --- |
| *Feeding* |  |  |  |  |  |  |  |  |  |  |  |
| Continued breastfeeding at ≥12 months | 52 (44%) | 119 | 73 (52%) | 140 | 6 (-9, 20)  *1.3 [0.7, 2.3]* | 64 (48%) | 133 | 4 (-12, 17)  *1.1* *[ 0.6, 2.1]* | 70 (43%) | 164 | -2 (-19, 10)  *0.8* *[0.5, 1.5]* |
| Introduction of cow’s milk | 34 (27%) | 127 | 38 (26%) | 145 | -1 (-14, 11)  *0.9 [0.5, 1.7]* | 31 (22%) | 142 | -6 (-20, 4)  *0.7* *[0.3, 1.3]* | 54 (30%) | 179 | 1 (-12, 12)  *0.9* *[0.5, 1.8]* |
| (CEBQ) |  |  |  |  |  |  |  |  |  |  |  |
| Food responsiveness | 2.5 (0.9) | 123 | 2.4 (0.8) | 144 | -0.1 (-0.3, 0.1) | 2.5 (0.8) | 140 | -0.1 (-0.3, 0.1) | 2.5 (0.9) | 174 | -0.1 (-0.3, 0.2) |
| Enjoyment of food | 4.2 (0.7) | 123 | 4.1 (0.8) | 144 | 0.0 (-0.2, 0.2) | 4.2 (0.7) | 140 | 0.0 (-0.2, 0.2) | 4.2 (0.7) | 174 | 0.0 (-0.2, 0.2) |
| Emotional over-eating | 1.7 (0.6) | 122 | 1.7 (0.7) | 144 | 0.0 (-0.1, 0.2) | 1.7 (0.6) | 139 | 0.0 (-0.2, 0.2) | 1.8 (0.7) | 174 | 0.0 (-0.2, 0.2) |
| Desire to drink | 2.7 (0.9) | 123 | 2.6 (0.9) | 144 | -0.2 (-0.5, 0.1) | 2.7 (1.0) | 140 | -0.1 (-0.3, 0.2) | 2.7 (1.0) | 174 | -0.2 (-0.4, 0.1) |
| Slowness in eating | 2.6 (0.6) | 123 | 2.7 (0.8) | 144 | 0.1 (-0.1, 0.3) | 2.7 (0.7) | 140 | 0.1 (-0.1, 0.3) | 2.8 (0.7) | 174 | 0.2 (0.0, 0.4) |
| Satiety responsiveness | 2.6 (0.7) | 123 | 2.8 (0.7) | 144 | 0.1 (-0.1, 0.3) | 2.9 (0.6) | 140 | 0.2 (0.0, 0.4) | 2.9 (0.7) | 174 | 0.1 (0.0, 0.3) |
| Emotional under-eating | 2.9 (1.0) | 123 | 3.0 (0.9) | 144 | 0.1 (-0.1, 0.4) | 2.9 (0.9) | 140 | 0.0 (-0.2, 0.3) | 3.1 (0.8) | 174 | 0.1 (-0.1, 0.4) |
| Food fussiness | 2.2 (0.7) | 123 | 2.2 (0.8) | 144 | 0.0 (-0.2, 0.2) | 2.2 (0.7) | 140 | 0.0 (-0.2, 0.2) | 2.2 (0.7) | 174 | 0.0 (-0.2, 0.2) |
| Food avoid subscale | 10.3 (2.3) | 123 | 10.8 (2.2) | 144 | 0.3 (-0.3, 1.0) | 10.7 (2.0) | 140 | 0.3 (-0.3, 0.9) | 11.0 (2.1) | 174 | 0.5 (-0.2, 1.1) |
| Food approach: avoid ratio | 0.9 (0.3) | 123 | 0.8 (0.3) | 144 | 0.0 (-0.1, 0.1) | 0.8 (0.2) | 140 | -0.1 (-0.2, 0.0) | 0.8 (0.3) | 174 | -0.1 (-0.2, 0.0) |

Data are mean (standard deviation) or number (percent). See Table 1 for oral glucose tolerance test diagnostic criteria associated with each group. Feeding variables assessed by the Child Feeding Questionnaire.^31^ CEBQ, Child Eating Behaviour Questionnaire. Analyses adjusted for potential confounding by maternal body size (BMI), socioeconomic status (New Zealand Deprivation Index), ethnicity and infant sex, with Dunnett correction of family-wise error.

# Table S3. Anthropometric measures at 9 months.

|  | Control | N | Group A  GDM by lower but not higher criteria: treated | N | Adjusted MD  (95% CI) | Group B  GDM by lower but not higher criteria: untreated | N | Adjusted MD  (95% CI) | Group C  GDM by higher criteria treated | N | Adjusted MD  (95% CI) |
| --- | --- | --- | --- | --- | --- | --- | --- | --- | --- | --- | --- |
| *Body size* |  |  |  |  |  |  |  |  |  |  |  |
| Length—cm† | 72.0 (2.8) | 51 | 71.9 (3.2) | 64 | -0.1 (-1.2, 1.3) | 71.0 (2.7) | 58 | -0.9 (-2.1, 0.4) | 71.5 (2.9) | 66 | -0.5 (-1.8, 0.7) |
| Weight—kg† | 9.09 (1.38) | 42 | 8.78 (1.33) | 52 | -0.40 (-0.98, 0.18) | 8.80 (1.13) | 38 | -0.35 (-0.97, 0.27) | 8.52 (1.14) | 48 | -0.63 (-1.26, 0.01) |
| BMI kg/m^2^ | 17.2 (2.1) | 38 | 17.0 (1.8) | 50 | -0.4 (-1.3, 0.5) | 17.3 (1.7) | 35 | 0.1 (-0.8, 1.1) | 16.8 (1.8) | 48 | -0.6 (-1.6,0.4) |
| Head circumference—cm† | 44.9 (1.8) | 48 | 44.6 (1.4) | 53 | -0.2 (-0.9,0.5) | 45.0 (1.6) | 54 | 0.4 (-0.4, 1.1) | 44.3 (1.8) | 52 | -0.3 (-1.1, 0.4) |
| *Z-scores for age and sex* |  |  |  |  |  |  |  |  |  |  |  |
| Length | 0.66 (1.25) | 51 | 0.51 (1.30) | 64 | - 0.06 (-0.61,0.50) | 0.22 (1.21) | 58 | -0.39 (-0.95, 0.17) | 0.30 (1.41) | 66 | -0.28 (-0.86, 0.30) |
| Weight | 0.56 (1.19) | 42 | 0.22 (1.24) | 52 | -0.37 (-0.93, 0.18) | 0.34 (1.07) | 38 | -0.26 (-0.85, 0.34) | -0.07 (1.20) | 48 | -0.58 (-1.18, 0.03) |
| BMI | 0.06 (1.38) | 38 | -0.07 (1.27) | 50 | -0.24 (-0.87, 0.39) | 0.14 (1.13) | 35 | 0.03 (-0.64, 0.70) | -0.27 (1.29) | 48 | -0.40 (-1.08, 0.28) |
| Head circumference | 0.52 (1.26) | 48 | 0.24 (1.10) | 53 | -0.18 (-0.72, 0.37) | 0.68 (1.08) | 54 | 0.26 (-0.28, 0.80) | 0.00 (1.40) | 52 | -0.27 (-0.84, 0.30) |

Data are mean (standard deviation). See Table 1 for oral glucose tolerance test diagnostic criteria associated with each group. † Measures taken by well child Providers (80%) and parents (20%). BMI, body mass index. Analyses adjusted for potential confounding by maternal body size (BMI), socioeconomic status (New Zealand Deprivation Index), ethnicity and infant sex, with Dunnett correction of family-wise error.

# Table S4. Feeding and nutritional intake at 9 months.

|  | Control | N | Group A  GDM by lower but not higher criteria: treated | N | Adjusted RD or MD  (95% CI)  *OR [95% CI]* | Group B  GDM by lower but not higher criteria: untreated | N | Adjusted RD or MD (95% CI)  *OR [95% CI]* | Group C  GDM by higher criteria treated | N | Adjusted RD or MD (95% CI)  *OR [95% CI]* |
| --- | --- | --- | --- | --- | --- | --- | --- | --- | --- | --- | --- |
| Feeding  Continued breastfeeding at ≥9months | 51 (57%) | 89 | 54 (60%) | 90 | 2 (-16, 20)  *1.1* *[0.5, 2.3]* | 57 (62%) | 92 | 4 (-13, 22)  *1.2 [0.6, 2.5]* | 59 (60%) | 99 | 2 (-16, 20)  *1.1 [0.5, 2.4]* |
| Introduction of cow’s milk | 6 (6%) | 96 | 6 (6%) | 95 | 1 (-20, 16)  *0.8 [0.2, 3.4]* | 7 (7%) | 99 | 1 (-20, 18)  *0.7 [0.2, 3.1]* | 8 (8%) | 104 | 2 (-20, 16)  *0.6 [0.1, 2.5]* |
| *CFFQ* |  |  |  |  |  |  |  |  |  |  |  |
| Energy—kJ/kg/day | 2845 (1755) | 86 | 2448 (1357) | 91 | -411  (-1004, 182) | 2475 (1687) | 93 | -419  (-1006, 168) | 2485  (1985) | 96 | -392  (-997, 213) |
| Macronutrients, percentage of energy |  |  |  |  |  |  |  |  |  |  |  |
| Protein | 15 (6) | 86 | 15 (7) | 91 | -0.1 (-2.2, 2.3) | 16 (7) | 92 | 1.6 (-0.7,3 .8) | 15 (6) | 96 | -0.6 (-1.8, 2.9) |
| Fat | 30 (10) | 86 | 30 (10) | 91 | -0.2 (-3.6, 3.1) | 30 (9) | 92 | 0.5 (-2.8, 3.8) | 29 (10) | 96 | -0.3 (-3.7,3.1) |
| Carbohydrate | 52 (9) | 86 | 53 (12) | 91 | 0.1 (-3.8, 3.9) | 51 (12) | 92 | -2.0 (-5.9, 1.8) | 54 (11) | 96 | 0.3 (-4.3, 3.6) |

Data are mean (standard deviation) or number (percent). See Table 1 for oral glucose tolerance test diagnostic criteria associated with each group. Complimentary Food Frequency Questionnaire. Analyses adjusted for potential confounding by maternal body size (BMI), socioeconomic status (New Zealand Deprivation Index), ethnicity and infant sex, with Dunnett correction of family-wise error.
